# Perioperative changes in osteopontin and TGFβ1 plasma levels and their prognostic impact for radiotherapy in head and neck cancer

**DOI:** 10.1186/s12885-016-3024-4

**Published:** 2017-01-03

**Authors:** Bülent Polat, Philipp Kaiser, Gisela Wohlleben, Thomas Gehrke, Agmal Scherzad, Matthias Scheich, Uwe Malzahn, Thomas Fischer, Dirk Vordermark, Michael Flentje

**Affiliations:** 1 Department of Radiation Oncology, University of Würzburg, Josef-Schneider-Straße 11, 97080 Würzburg, Germany; 2 Department of Oto-Rhino-Laryngology, Plastic, Aesthetic and Reconstructive Head and Neck Surgery, University of Würzburg, Würzburg, Germany; 3 Department of Epidemiology and Biostatistics, University of Würzburg, Würzburg, Germany; 4 Department of Radiation Oncology, University of Halle-Wittenberg, Halle, Germany

**Keywords:** Perioperative changes, Osteopontin, TGFβ1, Head and neck cancer, Survival

## Abstract

**Background:**

In head and neck cancer little is known about the kinetics of osteopontin (OPN) expression after tumor resection. In this study we evaluated the time course of OPN plasma levels before and after surgery.

**Methods:**

Between 2011 and 2013 41 consecutive head and neck cancer patients were enrolled in a prospective study (group A). At different time points plasma samples were collected: T0) before, T1) 1 day, T2) 1 week and T3) 4 weeks after surgery. Osteopontin and TGFβ1 plasma concentrations were measured with a commercial ELISA system. Data were compared to 131 head and neck cancer patients treated with primary (*n* = 42) or postoperative radiotherapy (*n* = 89; group B1 and B2).

**Results:**

A significant OPN increase was seen as early as 1 day after surgery (T0 to T1, *p* < 0.01). OPN levels decreased to base line 3-4 weeks after surgery. OPN values were correlated with postoperative TGFβ1 expression suggesting a relation to wound healing. Survival analysis showed a significant benefit for patients with lower OPN levels both in the primary and postoperative radiotherapy group (B1: 33 vs 11.5 months, *p* = 0.017, B2: median not reached vs 33.4, *p* = 0.031). TGFβ1 was also of prognostic significance in group B1 (33.0 vs 10.7 months, *p* = 0.003).

**Conclusions:**

Patients with head and neck cancer showed an increase in osteopontin plasma levels directly after surgery. Four weeks later OPN concentration decreased to pre-surgery levels. This long lasting increase was presumably associated to wound healing. Both pretherapeutic osteopontin and TGFβ1 had prognostic impact.

## Background

Head and neck cancer is one of the leading causes of cancer-related death with almost 60.000 new cases and 12.000 deaths per year in the US [1]. Standard treatment consists of primary surgery and adjuvant radiotherapy in locally advanced tumors. Concomitant chemo-radiotherapy is an alternative to surgery as a definitive treatment option [2]. Despite combined multimodality treatment survival rates at 5 years are still about 20–50% for stage III/IV tumors [3–5]. Modern treatment strategies try to elucidate specific molecular patterns and address these with novel therapeutics like EGFR directed antibodies or small molecules against growth factor receptors [6–8]. Identifying and targeting prognostic and predictive biomarkers is an attractive approach for the development of new treatment strategies.

One of these biomarkers is osteopontin (OPN). It is an actively secreted protein which can be detected in body fluids like blood or urine. Additionally it is overexpressed in many cancer types [9] and plays an important role in tumor progression [10]. Furthermore, it was shown that elevated plasma levels are associated with an unfavorable outcome in cancer [11–16]. High OPN levels are also correlated with tumor hypoxia which is a main resistance factor to radiation treatment [17, 18].

**Fig. 1:**
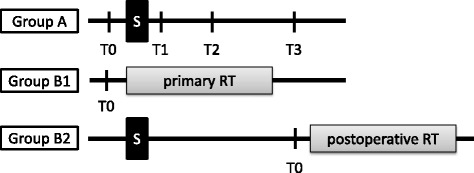
Scheme of the three patient groups treated by A) surgery, B1) definite radio-chemotherapy and B2) surgery followed by postoperative radiotherapy. Time points for blood samples are marked as T0 to T3 (T0, before surgery (group A) or before start of radiotherapy (group B1 and B2), T1, 1 day after surgery, T2, 1 week and T3, 3 to 4 weeks after surgery). S, surgery; RT, radiotherapy

Originally we compared OPN plasma levels in patients with head and neck cancer treated with definitive or postoperative radiotherapy. Surprisingly, there was no difference between both groups at the start of radiation treatment (data published as abstract) [19]. Therefore the osteopontin time course after primary surgery was analyzed in an additional cohort of head and neck cancer patients and data on prognostic significance have been updated. Expression patterns of TGFβ1 were studied in parallel to address the possible correlation of OPN plasma levels immediately after surgery with wound healing (see Fig. 1).

## Methods

### Patients and samples

Patients with newly diagnosed squamous cell carcinoma of the head and neck (HNSCC) were consecutively enrolled in two prospective trials (A and B1, 2). In group A we included patients with locally confined tumors which were eligible for primary resection. After giving their written informed consent, blood samples were taken at different time points: T0) before surgery, T1) 1 day after surgery, T2) 1 week and T3) 3 to 4 weeks after surgery. Blood samples were immediately centrifuged and plasma was stored at -80 °C. Group B1 consisted of patients who were medically or technically not eligible for surgical interventions or who refused surgery. In group B2 we recruited patients who were treated by primary surgery and were referred to adjuvant treatment according to their final tumor stage. Clinico-pathological patient characteristics are summarized in Table 1. Patients in group A were treated with primary surgery. According to national guidelines these patients received adjuvant treatment when appropriate. No adjuvant treatment was started before time point T3. In group B plasma samples of patients were analyzed before and during radio-(chemo) therapy (definitive treatment *n* = 41 (B1), postoperative treatment *n* = 89 (B2)). Patients from group B were enrolled before the start of the second trial (group A). The study was approved by the local clinical ethics committee. For a better understanding of the trial a scheme is shown in Fig. 1.

**Table 1:** Patient characteristics

	Group A: surgery	Group B1: primary RT	Group B2: postoperative RT	Controls	*p*-value
Number	41	42	89	16	
Time frame	08/11–09/13	07/07–06/09		09/07	
Follow-Up (median, months)	24.6	17.3	49.3^a^		**<0.01**
Gender m/f	34/7	37/5	70/19	8/8	n.s.
Age (mean)	62,3	61,0	59,9	41,6^a^	n.s.
T-stage					
1	9	0	27		**< 0.01**
2	15	1	28		
3	12	6	16		
4	4	34	17		
N-stage					
0	15	7	39		**< 0,01**
1	6	0	13		
2	20	29	35		
3	0	4	2		
UICC-stage					
I/II	5	1	31		**< 0,01**
III	14	1	12		
IV	22	40	46		
Tumor site					
Oropharynx	17	18	28		**0,004**
Larynx	10	11	18		
Hypopharynx	7	7	4		
Oral cavity	6	3	31		
CUP	1	3	8		

*Abbreviations*: *UICC* International union against cancer, *CUP* Cancer of unknown primary tumor. *P*-values according to student’s *t*-test and Fisher’s exact test

^a^Age was significantly lower in controls compared to patient groups

### Blood samples

Blood was anticoagulated with EDTA and subsequently centrifuged (4000 rpm) at room temperature for 10 min. Plasma was removed, aliquoted and stored at -80 °C until use. For comparison of OPN we used archived plasma samples collected from group B which had been prepared in the same way. These samples were collected just before the start of radiotherapy (T0).

### ELISA-OPN

Aliquots of each sample were analyzed in duplicate using the Human Osteopontin Assay Kit-IBL (Immuno-Biological Laboratories Co., Ltd., Japan) according to the manufacturer’s instructions.

### ELISA TGFβ1

The same aliquots were analysed in duplicate using a commercially available kit (ELISA Pro Kit for Human Latent TGFβ1, Mabtech, Sweden) according to the manufacturer’s instructions. Absolute plasma concentrations for osteopontin and TGFβ1 are given in ng/ml.

### Statistics

All statistical analyses were done with SPSS for Windows version 23.0 (IBM SPSS, Inc.). Statistical significance was set at p < 0.05. All reported *p* values were two-sided. For comparison of patient characteristics Fischer’s Exact test was used. Student’s *t*-test was used for comparison of plasma concentrations between groups. To test for correlations between plasma osteopontin and TGFβ1 we used Pearson product-moment correlation coefficient. Analysis of variance (ANOVA) was used for evaluation of OPN and TGFβ1 distribution among the different clinical parameters. For comparison of OPN and TGFβ1 values at different time points we employed a general linear model for repeated measures for each plasma marker. Kaplan-Meier analysis using log-rank statistics were used for comparing overall survival. As done in the DAHANCA 5 OPN sub-study [18] and TROG 02.02 study [20] groups were divided according to tertiles and median values of OPN and TGFβ1 concentrations.

## Results

### Patient characteristics

Table 1 describes the patient groups. Age and gender were comparable. Control patients were significantly younger. Patients from group B1 had more advanced tumor stages compared to group A and B2.

### Correlation of osteopontin and TGFβ1 with clinico-pathologic parameters

There was no association of OPN and TGFβ1 with clinical tumor parameters (e.g., histology, TNM- or UICC stage; data not shown).

### Osteopontin and TGFβ1 plasma levels

**Fig. 2:**
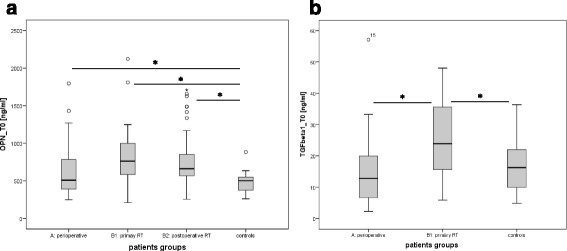
Box and whisker plots demonstrate the distribution plasma levels of **a**) OPN and **b**) TGFβ1 in the different patient groups and healthy controls at time point T0 before treatment. *Bars* indicate statistical significant differences with *p* < 0.05

Mean (±SD) osteopontin plasma concentration (ng/ml) was higher in patient groups compared to healthy controls (group A: 630.8 ± 353.0 ng/ml, group B1: 811.5 ± 365.1 ng/ml, group B2: 734.7 ± 310.1 ng/ml, controls: 478.9 ± 155.0 ng/ml; *p* = 0.028, *p* = 0.008 and *p* = 0.04 for group A, B1 and B2 vs controls, respectively, Fig. 2a). TGFβ1 plasma levels differed significantly between group A (15.23 ± 11.6 ng/ml) and group B1 (25.5 ± 11.8 ng/ml), *p* = 0.002 and between group B1 and controls (18.2 ± 10.1 ng/ml), p = 0.046 (Fig. 2b).

### Changes in osteopontin and TGFβ1 plasma concentrations over time after surgery

**Fig. 3:**
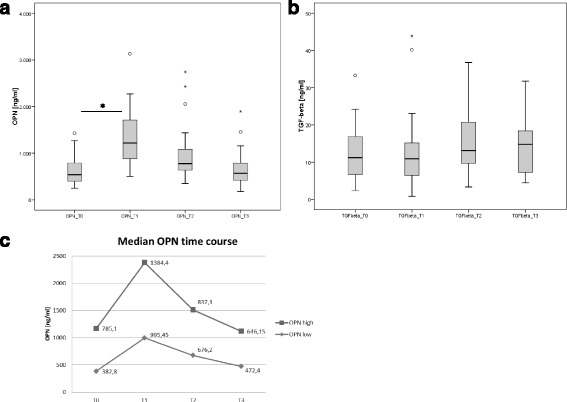
Time course of OPN plasma levels for group A with **a**) OPN and **b**) TGFβ1 (T0, before surgery, T1, 1 day after surgery, T2, 1 week and T3, 3 to 4 weeks after surgery). *Bars* indicate statistical significant differences with *p* < 0.05. **c** shows OPN time course for patients with OPN levels above or below median indicating that patients in both groups return to their pre-surgery status

Mean osteopontin plasma concentrations (ng/ml) for the different time points T0 to T3 (mean ± SD) was 631 ± 353, 1363 ± 660, 936 ± 526 and 649 ± 374, *p* < 0.01 (Fig. 3a). The most prominent difference was seen directly after surgery between time points T0 and T1. Three to four weeks after surgery OPN concentration reached base line levels again (T0 and T3). Patients with higher OPN concentrations (> median) at the time of surgery showed also higher values 3–4 weeks postoperatively (Fig. 3c, *p* < 0.05).

No significant changes were observed in the time course of TGFβ1 concentrations (Fig. 3b) with the highest TGFβ1 values at time points T2 and T3 (as we would expect it in wound healing).

### Correlation between osteopontin and TGFβ1

**Fig. 4:**
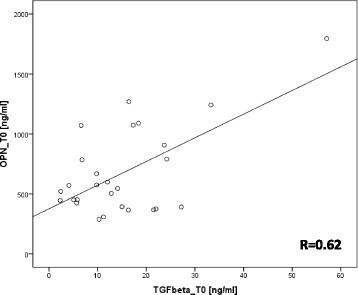
Positive correlation between TGFβ1 and OPN plasma levels at time point T0. Pearson correlation coefficient R = 0.619, *p* = 0.001

Pretherapeutic plasma concentrations of osteopontin and TGFβ1 values were analysed by the Pearson correlation coefficient test. We observed a significant positive correlation between both parameters, R = 0.619, *p* = 0.001 (Fig. 4).

### Survival

**Fig. 5:**
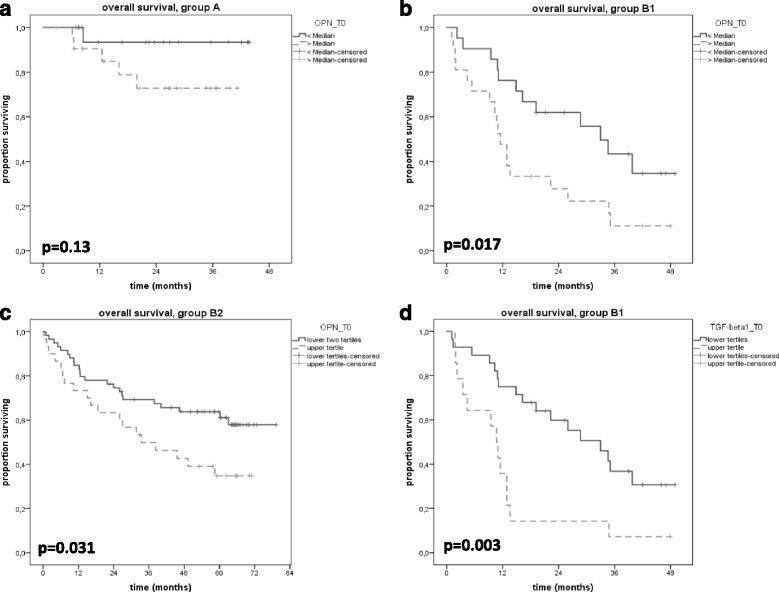
Kaplan-Meier curves show overall survival for patients in group **a** (perioperative, A), group B1 (primary radiotherapy, **b**) and group B2 (postoperative RT, **c**) according to OPN at time point T0. When dichotomized by median or tertiles, patients with lower OPN had an improved overall survival. For TGFβ1 a difference in survival was seen in patients from group B1, showing a better survival for patients in the lower two tertiles (**d**)

Both osteopontin and TGFβ1 at the start of treatment correlated with patient overall survival (Figs. 5a-d). Higher OPN values were associated with a shorter overall survival. Median survival times were 11.5 and 33.0 months, *p* = 0.017 in patients with definitive radiochemotherapy (group B1). Median survival was 33.4 months for patients with higher OPN values and was not reached for lower OPN values (*p* = 0.031) in patients treated with postoperative RT (group B2). In group A (patients with earlier tumor stage partly with no adjuvant treatment) survival was also worse for the high OPN group but the difference was not statistically significant (survival at 3 years was 76 and 95%, *p* = 0.13). Patients with TGFβ1 values in the upper tertile showed a worse outcome with median survival times of 10.7 and 33.0 months, *p* = 0.003 (group B1).

## Discussion

To our knowledge this is the first study presenting short term osteopontin expression after surgery in head and neck cancer patients. Blasberg and coworkers reported on OPN time course after tumor resection in lung cancer patients [21]. They described a similar pattern with decreasing OPN plasma values in the longer follow-up but did not study OPN changes within the first days and weeks after surgery.

Our results suggest that both tumor mass (related microenvironment) and the postsurgical situation can result in significantly elevated OPN levels. Instead of an anticipated immediate postoperative decrease we observed a doubling of OPN within 1 day and a return to preoperative values 3 to 4 weeks thereafter. Values at this time point seemed to mirror the situation before surgery. Adjuvant radiotherapy typically starts 4 weeks after surgery. Under the assumption that OPN is prognostic for malignant behavior and influences radiation response [22], this may explain that OPN before radiotherapy was prognostic both in primary and postoperative treatments.

### OPN and TGFβ1 in wound healing

We propose the hypothesis that the transitory rise in OPN plasma levels in the postoperative setting is associated with wound healing and not caused by OPN secretion or expression from cancer cells since its increase was seen within 24 h. It is well known that OPN is not a tumor specific protein and can also originate from immune cells like macrophages or from endothelial cells [23, 24]. In wound healing there is a wide range of cells and cytokines which are differentially expressed [25]. Therefore we chose TGFβ1 as a representative marker and looked for changes in its expression patterns. We observed an increase of its plasma concentration peaking at 1 week after surgery which is in line with data from the literature [26, 27]. Changes of OPN and TGFβ1 levels were correlated (R = 0.62). From preclinical studies there is good evidence for an OPN mediated TGFβ1 expression [28–30]. This is in agreement with the kinetics observed in this study, peak concentration of TGFβ1 lagged behind.

### TGFβ1 and OPN as prognostic factors

Transforming growth factor beta 1 is both expressed by tumor cells and adjacent stroma [31–33]. Prognostic impact of plasma levels is therefore controversial [34–38]. In this patient cohort we observed a significant negative correlation of pre-therapeutic TGFβ1 with overall survival.

The prognostic significance of osteopontin in head and neck cancer has been reported in patients treated by definite radiotherapy [15, 16, 18, 39] and is thought to relate to an association with tumor hypoxia and malignant phenotype. A hypoxic sensitizer (nimorazole) was of benefit in the high osteopontin tertile in the Dahanca 5 study. In contrast, data from TROG 02.02 did not find an association with survival parameters [20] and no predictive correlation with tirapazamine treatment.

Our data support a role of OPN as a prognostic biomarker for inoperable patients (treated with definite radiochemotherapy) and extend this observation to patients with combined surgery and radiotherapy.

Limitations of this and other single center studies are caused by the limited sample size. Furthermore, despite the fact that there is a large body of data on OPN detection there is still not a generally validated and certified test, making cross study comparisons more difficult. Most groups have been using an ELISA based system. But still there is also no standard ELISA kit, which would make at least these data more comparable. As shown by Vordermark et al. OPN values differed significantly when different ELISA systems were applied [40]. Also different OPN values are generated when using plasma or serum samples. For TGFβ1 the described ELISA system can only detect the total latent form and not the functionally active form of TGFβ1 which could also lead to some bias.

## Conclusion

In conclusion, patients with head and neck cancer showed a rise in osteopontin plasma levels as short as 24 h after surgery. Four weeks after tumor resection OPN concentration decreased to baseline levels mirroring the pre-treatment situation. This long lasting OPN increase was presumably associated with wound healing. Both osteopontin and TGFβ1 base line levels had prognostic impact on patient survival. Confirmation, especially for the postoperative setting as well as correlation with tumor gene signatures seems worthwhile.

## Abbreviations

EDTA: Ethylenediaminetetraacetic acid
ELISA: Enzyme-linked immunosorbent assay
HNSCC: Squamous cell carcinoma of the head and neck
OPN: Osteopontin
TGFβ1: Transforming growth factor 1
